# Puerarin Attenuates Diabetic Nephropathy by Promoting Autophagy in Podocytes

**DOI:** 10.3389/fphys.2020.00073

**Published:** 2020-02-14

**Authors:** Xueling Li, Qingqing Zhu, Rong Zheng, Jiayi Yan, Minggang Wei, Yichen Fan, Yueyi Deng, Yifei Zhong

**Affiliations:** ^1^Division of Nephrology, Longhua Hospital, Shanghai University of Traditional Chinese Medicine, Shanghai, China; ^2^The First Affiliated Hospital of Soochow University, Suzhou, China

**Keywords:** puerarin, diabetic nephropathy, podocyte, autophagy, apoptosis

## Abstract

Puerarin, an active compound of radix puerariae, is a major compound used in Chinese herbal medicines to treat patients with diabetic nephropathy (DN). In the previous studies, we showed that puerarin exerts renoprotective effects in Streptozocin (STZ)-induced diabetic mice through activation of Sirt1 and anti-oxidative effects. Here, we further investigated the underlying mechanism mediating the renal protective effects of puerarin in DN. We studied the effects and mechanism of puerarin in STZ-induced diabetic mice and in cultured immortalized mouse podocytes treated with high glucose. We confirmed that puerarin ameliorated urinary albumin creatinine ratio and kidney injury in STZ-induced DN mice. We found that expression of heme oxygenase 1 (HMOX-1) and Sirt1 was suppressed in diabetic glomeruli but restored by puerarin treatment at both mRNA and protein levels. Additionally, we found that puerarin induced autophagy in the kidney of DN mice. In conditionally immortalized mouse podocytes, puerarin inhibited HG-induced apoptosis and restored the mRNA and protein levels of HMOX-1 and Sirt1. Interestingly, we showed that puerarin decreased liver kinase B1 (LKB1) acetylation, thereby promoting adenosine 5′-monophosphate-activated protein kinase-dependent autophagy. Knockdown of HMOX-1 and Sirt1 expression or treatment with the autophagy inhibitor 3-methyladenine abolished the protective effects of puerarin in HG-treated podocytes. Taken together, these results suggest that puerarin protects podocytes from diabetes-induced injury through HMOX1 and Sirt1-mediated upregulation of autophagy, a novel mechanism explaining its renal protective effects in DN.

## Introduction

Diabetic nephropathy (DN) is a complication of diabetes and the leading cause of end stage renal disease worldwide (Yamahara et al., 2013). Glomerular damage and proteinuria, which are associated with diabetes, lead to the development of tubulointerstitial lesions and ultimately end-stage renal disease (Nath, 1992; Burton and Harris, 1996; Abbate et al., 2006). In the early stages, the development of which is associated with injury to podocytes (Nath, 1992). Podocytes are specialized kidney glomerulus cells that wrap around the capillaries and filter blood, preventing the entry of plasma proteins into the urinary filtrate (Reiser and Altintas, 2016). A reduction in podocytes density, which can be caused by apoptosis and detachment from the glomerular basement membrane, is a predictor of DN progression (Gunter et al., 2005; Weil et al., 2012).

Histone deacetylases are involved in the regulation of autophagy, an adaptive mechanism that is activated in response to stress. Autophagy mediates the removal of damaged proteins and organelles to maintain cellular homeostasis (Singh and Cuervo, 2011). During starvation, nuclear-localized Sirt1 was activated and deacetylated LC3 in the nucleus. Once the deacetylated molecules shift into the cytoplasm, they regulate autophagy by interacting with autophagic effectors such as Atf7 (Huang et al., 2015). Sirt1 deacetylates and activates LKB1, a serine-threonine protein kinase that phosphorylates and activates AMP-activated protein kinase (AMPK) (Lan et al., 2008). Sirt1 can also be activated by AMPK, and the AMPK-Sirt1 pathway plays important roles in diabetes, fatty liver disease, cancer, and cardiovascular diseases by modulating autophagy (Fukami et al., 2007; Cantó et al., 2010; Talero et al., 2016).

Autophagy, which plays a protective role against podocyte injury and loss in DN, is induced by heme oxygenase-1 (HMOX-1), an antioxidant enzyme that is induced in response to oxidative stress (Dong et al., 2015). HMOX-1 is upregulated in the kidney in response to various conditions associated with oxidative stress, such as toxin-induced nephropathy and ischemia-reperfusion injury (Lee et al., 2009). HMOX-1 promotes autophagy and inhibits apoptosis through the activation of AMPK (Dong et al., 2015).

In previous work from our group, we showed that puerarin, the active compound of *radix puerariae* and a traditional Chinese herbal medicine, exerts renoprotective effects in a mouse model of streptozotocin (STZ)-induced diabetes with endothelial nitric oxide synthase (eNOS) deficiency, which mimics the effects of advanced human DN (Li et al., 2017). In the present study, we expanded these results by investigating the role of autophagy in mediating the renoprotective effects of puerarin. The results showed that puerarin promoted autophagy and inhibited apoptosis through a mechanism involving the modulation of HMOX-1, Sirt1, and the activation of AMPK.

## Materials and Methods

### Experimental Animals

8-week old male mice on a C57BL/6 background were purchased from The Jackson Laboratory (Bar Harbor, ME, United States). The animal room was maintained at a temperature of 22 ± 2°C with a 12 h light–dark cycle (6:00–18:00) and 65 ± 5% humidity. All animals received humane care in compliance with the institutional animal care guidelines approved by the Experimental Animal Ethical Committee, Shanghai University of Traditional Chinese Medicine. Diabetes was induced by intraperitoneal injection of freshly prepared STZ (Sigma-Aldrich, St. Louis, MO, United States) dissolved in 0.1M citrate buffer, pH 4.5 at 50 mg/kg for 5 consecutive days (Li et al., 2017). Blood glucose was measured every week with Abbott blood glucometer (Abbott, Alameda, CA, United States), the induction of diabetes was considered successful when blood glucose > 16.6 mmol/L after 2 weeks. The mice, which developed significant albuminuria, were considered as the successful model for DN. In the study, 5 mice failed to reach the blood glucose standard for diabetes and 3 mice failed to develop significant albuminuria. These mice were excluded from the experimental groups. The mice were given puerarin (Sigma-Aldrich) dissolved in 5% DMSO by oral gavage at different doses or 5% DMSO vehicle as control for 12 weeks. Fifty-six mice were randomly divided into seven groups (*n* = 8) and fed and treated as follows: Group 1: control mice received DMSO. Group 2: control mice received puerarin at 40 mg/kg, Group 3: STZ mice received DMSO, Group 4: STZ mice received puerarin 5 mg/kg, Group 5: STZ mice received puerarin 10 mg/kg, Group 6: STZ mice received puerarin 20 mg/kg, and Group 7: STZ mice received puerarin 40 mg/kg. Urine samples were collected every week until mice were sacrificed. Urine albumin levels were quantified by using commercial kits obtained from Nanjing Biotechnology, Co., Ltd. (Nanjing, China). Anesthetized mice were euthanized by cardiac puncture and blood withdrawal. Immediately after cardiac puncture, the kidneys were harvested. A portion of fresh kidney tissue was fixed in 10% buffered formalin, and the remaining tissue was snap frozen in liquid nitrogen and stored at −80°C.

### Kidney Histology

Samples of kidney tissue were fixed in 10% phosphate buffered saline (PBS)-formalin for at least 24 h and then embedded in paraffin for histological assessment. Sections of samples were used for immunostaining for periodic acid-Schiff (PAS) or other markers. They were then examined under a light microscope (Olympus, Japan).

### Immunofluorescence Staining

Indirect immunofluorescence staining was performed according to an established procedure. Briefly, cells cultured on coverslips were washed twice with cold PBS and fixed with cold methanol/acetone (1:1) for 10 min at −20°C. Following three extensive washings with PBS, the cells were treated with 0.1% Triton X-100 for 5 min and blocked 2% normal donkey serum in PBS buffer for 1 h at room temperature and then incubated with the specific primary antibodies against LC3 and Laminin, followed by staining with FITC or TRITC-conjugated secondary antibody. Cells were stained with 49,6- diamidino-2-phenylindole (DAPI) (Invitrogen) to visualize the nuclei. Slides were viewed with a Nikon Eclipse 80i Epifluorescence microscope equipped with a digital camera (DSRi1, Nikon). In each experimental setting, immunofluorescence images were captured with identical light exposure times.

### Immunohistochemistry

Samples were deparaffinized with xylol and then sliced into 4-μm sections. Sections were rehydrated using a graded ethanol series. A heat-induced epitope protocol was used for antigen-retrieval (95°C for 40 min). Samples were incubated in methanol containing 0.3% hydrogen peroxide to block endogenous peroxidases. Samples were pre-incubated with serum (Vectastain Elite ABC kit; Vector Laboratories, Inc., Burlingame, CA, United States) and then incubated (overnight at 4°C) with polyclonal rabbit anti-mouse HMOX-1 antibody at 1:1000 (Abcam, Shanghai, China, ab13248). After washing three times in TBST (150 mM NaCl, 10 mM Tris-HCl, pH 7.6), sections were incubated with secondary antibody for 20 min at room temperature. Peroxidase-conjugated biotin-streptavidin complex (Dako, Glostrup, Denmark) was then applied to the sections for 20 min. Sections were visualized with 3, 3′-diaminobenzidine and counterstained with hematoxylin. The negative control used non-immune serum instead of primary antibody.

### Cell Culture

Conditionally immortalized mouse podocytes were obtained from the Cell Bank at the Chinese Academy of Sciences (Shanghai, China) and cultured in DMEM containing 10% fetal calf serum.

### Cell Transfection

For HMOX-1 and Sirt1 knockdown. si-HMOX-1 and si-Sirt1 vector were constructed by GenePharma (Shanghai, China), and podocytes were transfected with either si-HMOX-1 or si-Sirt1 at a final concentration 50 nM using Lipofectamine 2000 (Invitrogen, Carlsbad, CA, United States) according to the manufacturer’s protocol.

### Flow Cytometry Assay

Logarithmically growing podocytes were seeded into culture flasks. The cells were dual stained with Annexin V-FITC and propidium iodide (PI) for 30 min at room temperature. The stained cells were immediately analyzed by flow cytometry (Becton Dickinson, Franklin Lakes, NJ, United States). Apoptotic cells were defined as Annexin V-FITC positive and PI negative.

### RNA Isolation and Quantitative Real-Time PCR

RNA was isolated using the TRIzol reagent (Invitrogen) according to the manufacturer’s instructions in podocytes and mouse kidney samples and reverse transcribed using a miScript Reverse Transcription kit (Qiagen). QRT-PCR was performed using the SYBR Premium Ex Taq II kit (Takara, Dalian, China) in an ABI PRISM 7500 Sequence Detection System (Applied Biosystems). All reactions were performed in triplicate and the mean value was used to calculate expression levels after normalization to β-actin as an internal standard. The primer sequences used in this study are listed in Table 1.

**TABLE 1:** Sequences of primers for quantitative real-time PCR.

	Gene name	Forward primer sequence (5′→ 3′)	Reverse primer sequence (5′→ 3′)
Mouse	HMOX1	CAGAAGAGGCTAAGACCGCC	TCTGACGAAGTGACGCCATC
	Sirt1	ATACGGAGAGGCCCGAATGT	ATACGGAGAGGCCCGAATGT
	β-Actin	TTCGTTGCCGGTCCACACCC	GCTTTGCACATGCCGGAGCC

### Immunoprecipitation

For immunoprecipitation studies, 5 μg of anti-LKB1 or Rabbit IgG-AC (Proteintech 10746-1-AP: Santa Cruz, Dallas, TX, United States, ab37415) was added to cell lysates and incubated overnight at 4°C, under constant rotation. Immune complexes were precipitated and washed in lysis buffer. Immunoprecipitated samples were subject to western blotting analysis by using anti-acetylation antibody (Santa Cruz, Dallas, TX, United States, ab51997) and anti-LKB1 antibody.

### Protein Extraction and Western Blot Analysis

Podocytes were lysed using RIPA buffer, and protein concentration was determined using the BCA protein assay kit. Approximately 30 μg of protein from each sample was separated using a 10% SDS-polyacrylamide gel and transferred to PVDF membranes. Membranes were blocked with 5% skim milk in TBST and incubated with primary antibodies overnight at 4°C. Membranes were then incubated with the corresponding secondary antibodies for 1 h at room temperature and washed in TBST. Proteins were detected using specific antibodies: HMOX1: Abcam, ab13248; Sirt1: Abcam,ab110304; LC3B:Abcam,ab63817; AMPK:Abcam,ab110036; p-AMPK: Abcam,ab194920; P62: Abcam,ab56416; beta-actin: Abcam: ab115777.

### Transmission Electron Microscopy

Cells were fixed with 2.5% glutaraldehyde in phosphate buffer and stored at 4°C until embedding. Cells were post-fixed with 1% osmium tetroxide followed by an increasing gradient dehydration step using ethanol and acetone. Cells were then embedded in Araldite, and ultrathin sections were obtained (50–60 nm), placed on uncoated copper grids, and stained with 3% lead citrate–uranyl acetate. Images were examined with a CM-120 electron microscope (Philips).

### Statistical Analysis

Statistical analyses were performed with SPSS 17.0 software. The statistical significance of differences was determined by either the Student’s *t*-test for comparison between means or one-way analysis of variance. Data were considered statistically significant at *P* < 0.05.

## Results

### Puerarin Treatment Ameliorates Kidney Injury in DN Mice

**FIGURE 1:**
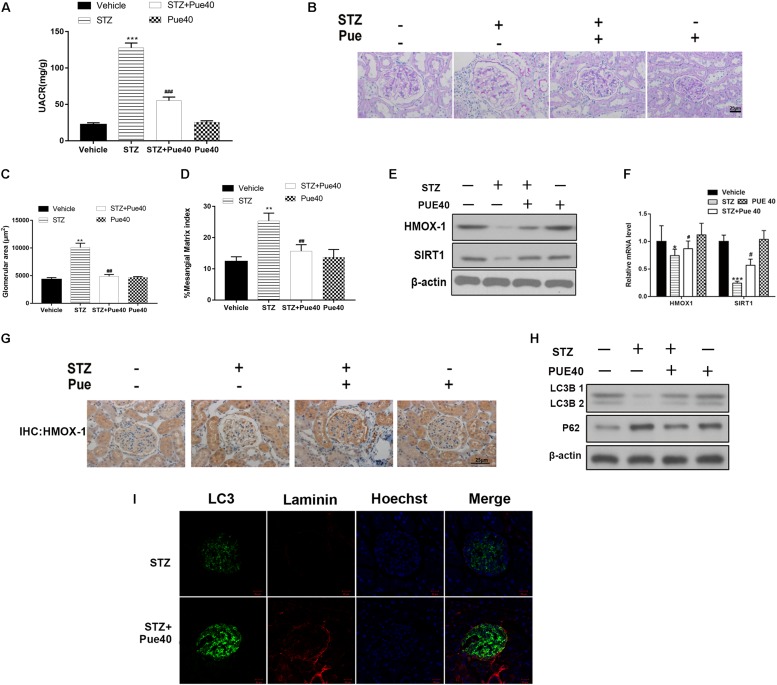
Puerarin ameliorates kidney injury in mice with streptozocin (STZ)-induced diabetic nephropathy (DN). **(A)** UACR were detected at 12 weeks after STZ injection. **(B)** Representative images of periodic acid-Schiff (PAS) stained kidney sections. **(C)** Glomerular area quantification is shown at 12 weeks after STZ injection. **(D)** The percentage of mesangial matrix area in the glomeruli at 12 weeks after STZ injection. **(E)** Western blot analysis of the expression of HMOX-1, and Sirt1 in the kidney. **(F)** RT-PCR analysis of the mRNA expression of HMOX-1, and Sirt1 in the kidney. **(G)** Representative images of immunohistochemical staining for HMOX-1 in kidney sections. **(H)** The kidney protein expression of LC3B and p62 were determined by western blotting. **(I)** Kidney section were immunostained with anti-LC3 antibody (green) to identify autophagosomes, followed by staining with anti-Laminin antibody (red) to sever as a marker for the basement membrane and anti-Hoechst 33342 antibody (blue). Data are expressed as the mean ± SEM (*n* = 8). *P < 0.05; **P < 0.01; ***P < 0.001 vs. control; #*P* < 0.05; ##*P* < 0.01; ###*P* < 0.001 vs. STZ.

Mice with STZ-induced diabetes, which was confirmed by blood glucose levels > 250 mg/dl, and control mice were treated with puerarin (40 mg/kg) or DMSO. UACR was considered as a hallmark for DN (Williams, 2005), increased in diabetic mice, Puerarin reduced the UACR levels after 12 week treatment in diabetic mice (Figure 1A). Morphological changes in the kidney of STZ-induced diabetic mice were observed (Figure 1B). The glomerular area and mesangial matrix area were significantly increased in STZ-treated mice, treatment with puerarin restored the values to the control levels (Figures 1C,D). The expression of HMOX-1 and Sirt1 was assessed by western blotting of mouse kidneys. The results showed that STZ significantly downregulated HMOX-1 and Sirt1 expression, and treatment with puerarin restored the expression of these proteins to the control levels (Figure 1E). Consistently, qPCR analysis of the mRNA expression of these molecules showed that puerarin significantly upregulated HMOX-1 and Sirt1 in the diabetic kidney (Figure 1F). Immunohistochemical staining showed that puerarin also restored the expression of HMOX-1 in the diabetic kidney (Figure 1G). The effect of puerarin on autophagy was determined by western blotting analysis of kidney cortices for autophagy markers. The results showed that puerarin significantly restored the expression of the autophagosomal marker LC3B and downregulated p62 protein levels in the STZ-treated mice (Figure 1H). Consistent with this, the staining of LC3 was also increased in the diabetic mice treated with puerarin. Formation of autophagosomes in kidneys were examined by immunofluorescence staining of LC3. As shown in Figure 1I, the distribution of the autophagosomes (stained with the anti-LC3 antibody, green) were localized mainly on the epithelial side of the glomerular basement membrane (stained with the anti-Laminin antibody, red) where the podocytes reside. Immunofluorescence staining illustrated the diminished staining of LC3, coupled with the thickened staining of laminin in diabetic glomeruli, indicating that autophagy was induced primarily by puerarin. These results indicated that puerarin induces autophagy in the kidney of DN mice.

### Puerarin Protects From High Glucose (HG)-Induced Apoptosis in Podocytes

**FIGURE 2:**
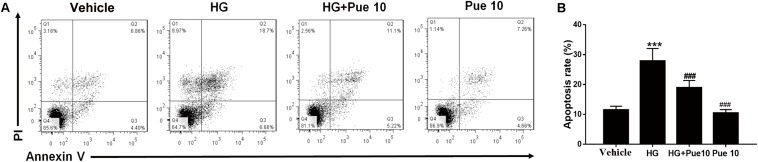
Puerarin protects from high glucose (HG)-induced injury in podocytes. Cells were pre-incubated with puerarin for 15 min, and then incubated with HG (30 mM) for 48 h. **(A,B)** Analysis of apoptosis rates by flow cytometry and quantification. Data are expressed as the mean ± SEM (*n* = 3). ***P < 0.001 vs. control; ###*P* < 0.001 vs. HG.

Loss of or injury to podocytes is characteristic of DN, and HG conditions induce apoptosis of podocytes (Katalin et al., 2006). We therefore examined the effect of puerarin in immortalized mouse podocytes exposed to HG. Flow cytometric analysis of apoptosis showed that puerarin significantly reversed HG-induced apoptosis in podocytes (Figures 2A,B).

### Puerarin Promotes Autophagy in Podocytes

**FIGURE 3:**
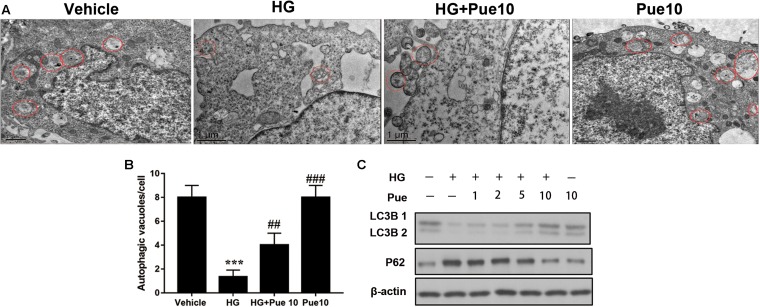
Puerarin stimulated autophagy in podocytes. **(A,B)** Autophagic vacuoles (autophagosomes) were detected by transmission electron microscopy (TEM). Representative TEM images are shown and autophagosomes are marked with red arrows. The number of autophagosomes per cell was calculated by counting the number of double-membrane organelles in 10 cells. **(C)** The protein expression of LC3B and p62 in podocytes was determined by western blotting. Data are expressed as the mean ± SEM (*n* = 3). ***P < 0.001 vs. control; ##*P* < 0.01; ###*P* < 0.001 vs. HG.

To further examine the involvement of autophagy in the protective effect of puerarin, autophagosomes were detected by transmission electron microscopy (TEM). The results showed that HG decreased the number of autophagosomes per cell, and puerarin significantly restored the formation of autophagosomes in podocytes (Figures 3A,B). To confirm the involvement of autophagy, cell lysates were analyzed for the expression of the autophagy markers LC3B and p62. The results of western blotting showed that puerarin restored the HG-induced downregulation of LC3B and upregulation of p62 at the protein levels (Figure 3C). Taken together, these results indicated that puerarin reverse autophagy inhibition induced by high glucose *in vitro*.

### Puerarin Modulated the Effect of High Glucose (HG) on HMOX1/SIRT1

**FIGURE 4:**
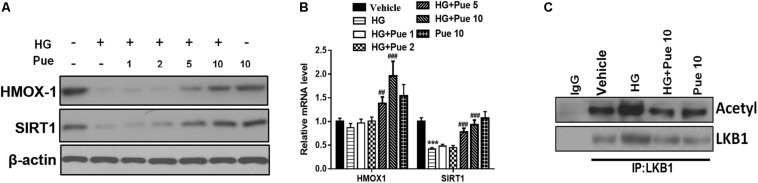
Puerarin modulated the effect of high glucose (HG) on HMOX1/SIRT1. **(A)** The expression of HMOX-1 and Sirt1 in podocytes was determined by western blotting. **(B)** The mRNA expression of HMOX-1 and Sirt1 was determined by RT-PCR. **(C)** Whole cell extracts were immunoprecipitated with LKB1 antibody or an equal amount of rabbit IgG and blotted with acetylated-lysine (Acetyl) antibody and anti-LKB1 antibody. ***P < 0.001 vs. control; ##*P* < 0.01; ###*P* < 0.001 vs. HG.

The results of western blotting and qPCR showed that puerarin significantly reversed the HG-induced downregulation of HMOX-1, and Sirt1 in a dose dependent manner (Figures 4A,B). To investigate the underlying mechanisms, podocyte lysates were immunoprecipitated using an anti-LKB1 antibody and immunoblotted against acetylated-lysine to examine the potential involvement of AMPK. The results showed that puerarin partially reversed the HG induced acetylation of LKB1 (Figure 4C), suggesting that the protective effect of puerarin could be mediated by the modulation of LKB1 acetylation via the AMPK/Sirt1 axis.

### Puerarin Protects Against HG-Induced Podocyte Injury and Apoptosis via HMOX-1/Sirt1

**FIGURE 5:**
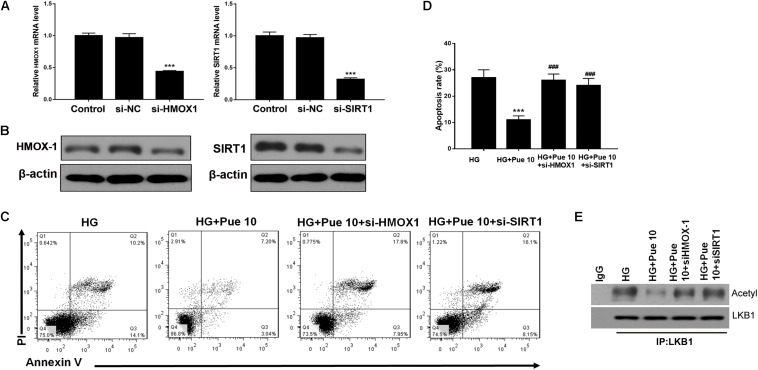
Puerarin protected against HG-induced injury in podocytes via HMOX-1/Sirt1. Cells were pre-incubated with puerarin for 15 min, and then incubated with HG (30 mM) for 48 h. **(A)** The mRNA expression of HMOX-1, and Sirt1 in podocytes without HG or Puerarin was determined by RT-PCR. **(B)** The expression of HMOX-1 and Sirt1 in podocytes without HG or Puerarin was determined by western blotting. **(C,D)** Analysis of apoptosis rates by flow cytometry and quantification. Data are expressed as the mean ± SEM (*n* = 3). **(E)** Whole cell extracts were immunoprecipitated with LKB1 antibody or an equal amount of rabbit IgG and blotted with acetylated-lysine (Acetyl) antibody and anti-LKB1 antibody. ***P < 0.001 vs. HG; ###*P* < 0.001 vs. HG + Pue 10.

The mechanisms underlying the effect of puerarin were further investigated by silencing the expression of HMOX-1 and Sirt1 in podocytes stimulated with HG. First, we examined the efficacy of silencing in untreated cells by western blotting and RT-PCR (Figures 5A,B). Flow cytometric analysis showed that knockdown of HMOX-1 and Sirt1 inhibited the effect of puerarin on decreasing HG-induced apoptosis in podocytes (Figures 5C,D). Immunoprecipitation of lysates from HG-stimulated podocytes showed that knockdown of HMOX-1 and Sirt1 abolished the effect of puerarin on decreasing the HG-induced acetylation of LKB1 (Figure 5E). Taken together, these results confirmed that the protective effect of puerarin against podocyte injury and apoptosis was mediated by the HMOX-1/Sirt1 axis and the modulation of AMPK activity.

### Puerarin Restores Autophagic Activity in Podocytes via HMOX-1/Sirt1

**FIGURE 6:**
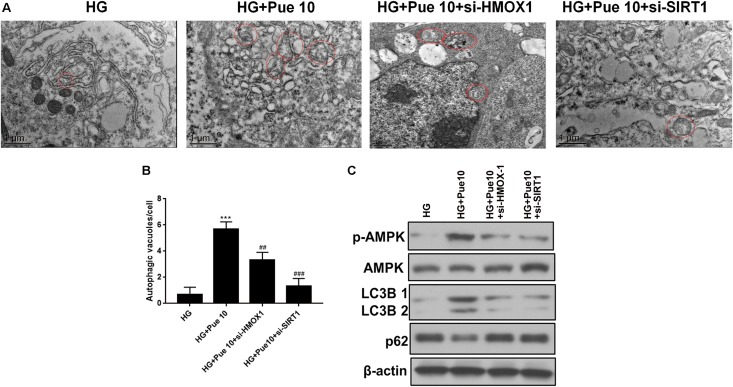
Puerarin stimulated autophagy in podocytes via HMOX-1/Sirt1. **(A,B)** Autophagic vacuoles (autophagosomes) were detected by TEM. Representative TEM images are shown and autophagosomes are marked with red arrows. The number of autophagosomes per cell was calculated by counting the number of double-membrane organelles in 10 cells. **(C)** The protein expression of LC3B, p62, AMPK, and p-AMPK in podocytes was determined by western blotting. Data are expressed as the mean ± SEM (*n* = 3). ***P < 0.001 vs. control; ##*P* < 0.01; ###*P* < 0.001 vs. HG.

The effect of HMOX-1 and Sirt1 silencing on the protective effect of puerarin mediated by increased autophagy was further investigated. HMOX-1 and Sirt1 knockdown abolished the puerarin-induced restoration of autophagosome formation in podocytes exposed to HG (Figures 6A,B). Western blot analyses of autophagy markers showed that the effect of puerarin on restoring the expression of LC3B and downregulating p62 was abolished by HMOX-1 and Sirt1 knockdown (Figure 6C). In addition, western blot analysis of phospho-AMPK showed that silencing of HMOX-1 and Sirt1 inhibited the puerarin induced upregulation of p-AMPK and therefore the puerarin-induced restoration of AMPK activity in HG-treated podocytes (Figure 6C). These results confirmed that puerarin protects against podocyte injury by restoring the autophagic activity via the AMPK/Sirt1 pathway.

### Autophagy Modulation Mediates HG-Induced Podocyte Injury and the Protective Effect of Puerarin

**FIGURE 7:**
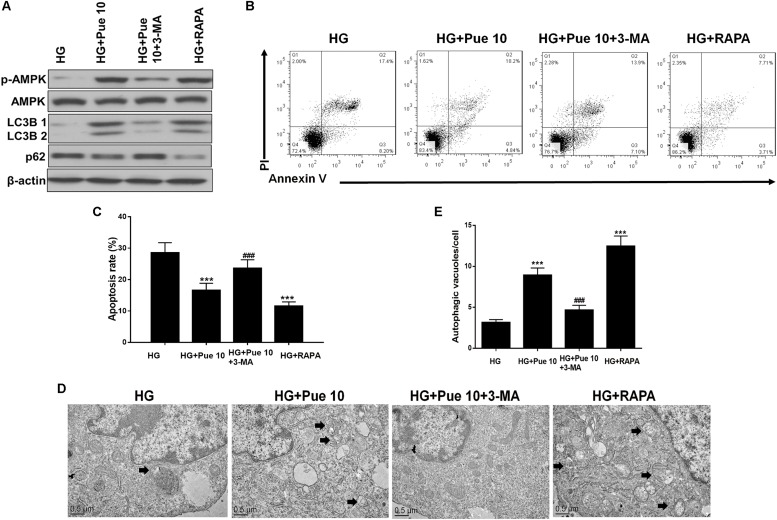
Autophagy is important for HG-induced podocyte injury. **(A)** The protein expression of p-AMPK, AMPK, LC3B, and p62 in podocytes was determined by western blotting. **(B,C)** Analysis of apoptosis rates by flow cytometry and quantification. **(D,E)** Autophagic vacuoles (autophagosomes) were detected by TEM. Representative TEM images are shown and autophagosomes are marked with black arrows. The number of autophagosomes per cell was calculated by counting the number of double-membrane organelles in 10 cells. Data are expressed as the mean ± SEM (*n* = 3). ***P < 0.001 vs. control; ###*P* < 0.001 vs. HG.

The role of autophagy in HG-induced podocyte injury and in the protective effect of puerarin was confirmed by treating cells with the autophagy inhibitor 3-MA or the autophagy inducer rapamycin. Rapamycin treatment restored the HG-induced downregulation of LC3B and upregulation of p62 in podocytes in a similar way to the effect of puerarin (Figure 7A). In addition, the results of western blot analysis showed that 3-MA abolished the effect of puerarin on restoring p-AMPK expression, whereas rapamycin had the opposite effect (Figure 7A). Inhibition of autophagy abolished the effect of puerarin on decreasing HG-induced apoptosis, whereas rapamycin inhibited apoptosis in a similar way to the effect of puerarin (Figures 7B,C). TEM analysis showed that 3-MA abolished the effect of puerarin on inducing autophagosome formation in HG-treated cells, whereas rapamycin promoted autophagosome formation in HG-treated podocytes (Figures 7D,E). Taken together, these results indicated that inhibition of autophagy exacerbated HG-induced podocyte injury and abolished the protective effect of puerarin, whereas autophagy induction by rapamycin mimicked the effects of puerarin, confirming that puerarin protects podocytes against injury by modulating autophagy.

## Discussion

In our previous study, we showed that puerarin exerts renoprotective effects by upregulating Sirt1 and activating Sirt1-mediated NF-κB deacetylation, thus protecting against oxidative damage in a mouse model of DN (Li et al., 2017). In the present study, we examined the mechanisms underlying the protective effects of puerarin by investigating the role of autophagy using a mouse model of DN and podocytes exposed to HG conditions. The results showed that the renoprotective effects of puerarin are mediated by the restoration of autophagic activity through the AMPK/Sirt1 pathway, providing additional support for the use of puerarin for the treatment of DN.

Autophagy seems to be a well-established contributor to podocyte homeostasis, and either enhanced (Cinà et al., 2012) or dysregulated autophagy (Björn et al., 2010) might involve in podocyte injury. In the present study, the effect of puerarin on ameliorating STZ-induced kidney injury was accompanied by the restoration of the expression of autophagy markers in the kidney, indicating the involvement of autophagy. The effect of puerarin on autophagy was confirmed in the podocytes exposed to HG conditions, in which puerarin restored Sirt1 expression, reversed the HG induced acetylation of LKB1, and restored autophagosome formation and the expression of autophagy markers. Autophagy exerts protective effects against renal damage associated with aging, hypoxia, and anticancer drugs (Hartleben et al., 2010; Kume et al., 2010; Kimura et al., 2011; Takahashi et al., 2012). Autophagy is also involved in the maintenance of podocyte function, as suggested by the high rates of autophagy in podocytes and the effect of depletion of autophagy-related proteins on glomerulopathy in mice (Hartleben et al., 2010). The role of autophagy in DN was suggested previously, as HG conditions inhibit autophagy in cultured podocytes, and inhibition of autophagy impairs podocyte function (Fang et al., 2013). Autophagy is regulated by nutrient state and intracellular stress, which are altered under diabetic conditions, potentially exacerbating organelle dysfunction and leading to DN by interfering with autophagy (Yamahara et al., 2013). These findings indicate that hyperglycemia-induced alterations in autophagic activity is a key mechanism underlying diabetes-related podocyte injury.

In the present study, we showed that puerarin partially reversed the HG-induced acetylation of LKB1, which activates AMPK. In addition, silencing of HMOX-1 and Sirt1 decreased HG-induced LKB1 acetylation and apoptosis, indicating that puerarin exerts its protective effects via HMOX-1 and Sirt1. Furthermore, HMOX-1 and Sirt1 knockdown inhibited the puerarin-induced upregulation of p-AMPK in HG-treated podocytes. These results confirmed that puerarin protects against podocyte injury by promoting autophagy via the HMOX-1, Sirt1, and AMPK pathway. Autophagy is activated in response to nutrient deprivation, and two signaling cascades are known to sense nutrient status and modulate autophagy: the mammalian target of rapamycin complex 1 (mTORC1), which inhibits autophagy, and the AMPK pathway, which induces autophagy (Steinberg and Kemp, 2009; Zoncu et al., 2011). AMPK activation reduces podocyte permeability to albumin and podocyte dysfunction in STZ-induced DN in mice (Sharma et al., 2008), supporting that AMPK activation exerts a protective effect on podocytes. We showed that treatment with 3-MA or rapamycin to inhibit or promote autophagy affected the protective effect of puerarin, further supporting that puerarin modulates autophagy to have renal protective effects. Published studies showed that HMOX-1 positively regulates SIRT1 signal transduction, thereby inhibiting macrophage activation (Nakamura et al., 2017). In a high-fat mouse model, the expression of Sirt1 in liver tissue was induced by administration of cobalt protoporphyrin, an active inducer of hmox-1 (Liu et al., 2015). These data also suggest that HMOX-1 can regulate Sirt1 expression and activity. Our current data suggest that Sirt1 regulates LKB1 acetylation. Therefore, HMOX1 may prevent LKB1 acetylation through regulation of Sirt1. It has been also reported that SIRT1 can induce phosphorylation of AMPK to activate autophagy (Price et al., 2012; Ganesan et al., 2017). Song et al. (2017) showed that puerarin can activate AMPK-mTOR pathway in renal epithelial cells to promote autophagy. Therefore, we speculate that puerarin stimulates SIRT1 expression in podocytes to deacetylate LKB1 and then phosphorylate AMPK-mTOR pathway to induce autophagy.

We believe that the role of puerarin is not limited to the treatment of DN or podocyte injury. Since puerarin has anti-oxidative stress effect and regulates autophagy, it should have protective effects in other kidney cell types too. In fact, puerarin also plays an important role in the treatment of many other diseases. Puerarin has beneficial effects on osteoporosis, neurological disorders, and cardiovascular diseases (Wong and Rabie, 2007; Lin et al., 2012;Li et al., 2016; Hou et al., 2018). The role of autophagy in mediating the effect of puerarin was reported previously for other diseases. Li and colleagues showed that puerarin increases survival, reduces ALT and AST levels, decreases the production of pro-inflammatory cytokines, and suppresses apoptosis in mice with lipopolysaccharide-D-galactosamine induced liver injury by promoting autophagy and inhibiting apoptosis (Li et al., 2018). In an *in vitro* model of anoxia/reoxygenation injury in primary cardiomyocytes, puerarin attenuates injury by promoting autophagy and decreasing apoptosis (Ma et al., 2016). However, Wang et al. (2018) showed that puerarin protects against ischemia/reperfusion injury in the brain by inhibiting autophagy through the activation of AMPK-mTOR-ULK1 signaling, which is not consistent with the present results. Therefore, the regulatory effect of puerarin on autophagy could be tissue or cell-specific. In DN, in addition to autophagy, several other mechanisms have been proposed for its renoprotective effects such as anti-oxidant and antiapoptotic effects (Yifei et al., 2014; Zhang et al., 2015; Song et al., 2016; Xu et al., 2016). Additional studies are necessary to clarify the contribution of each mechanism in mediating the renal protective effect of puerarin and how they interact each other.

## Conclusion

The present study explored the mechanisms underlying the protective effect of puerarin in diabetic mice with DN and in podocytes exposed to diabetic condition. The results showed that puerarin ameliorates kidney injury by restoring autophagic activity and suppressing apoptosis through a pathway likely involving the modulation of the HMOX-1, Sirt1, and AMPK. These results provide new insights into the renal protective mechanism of puerarin in human with DN.

## Data Availability Statement

The datasets generated for this study are available on request to the corresponding author.

## Ethics Statement

The animal study was reviewed and approved by Experimental Animal Ethical Committee, Shanghai University of Traditional Chinese Medicine.

## Author Contributions

XL and QZ carried out experiments and wrote the manuscript. RZ, JY, MW, and YF helped to analyze experimental results. YD and YZ designed experiments and amend the manuscript.

## Conflict of Interest

The authors declare that the research was conducted in the absence of any commercial or financial relationships that could be construed as a potential conflict of interest.
